# Treatment of Multisystem Inflammatory Syndrome in Children: Understanding Differences in Results of Comparative Effectiveness Studies

**DOI:** 10.1002/acr2.11478

**Published:** 2022-06-27

**Authors:** Michael Melgar, Eleanor G. Seaby, Andrew J. McArdle, Cameron C. Young, Angela P. Campbell, Nancy L. Murray, Manish M. Patel, Michael Levin, Adrienne G. Randolph, Mary Beth F. Son, BATS Consortium and the Overcoming COVID‐19 Investigators

**Affiliations:** ^1^ COVID‐19 Response Team, Centers for Disease Control and Prevention Atlanta Georgia; ^2^ Imperial College London, London, UK, University of Southampton, Southampton, UK, and Broad Institute of Massachusetts Institute of Technology and Harvard Cambridge; ^3^ Imperial College of London London UK; ^4^ Boston Children's Hospital Boston Massachusetts; ^5^ COVID‐19 Response Team, Centers for Disease Control and Prevention, Atlanta, Georgia, and Commissioned Corps of the US Public Health Service Rockville Maryland; ^6^ Imperial College Healthcare National Health Service Trust and Imperial College London London UK; ^7^ Harvard Medical School and Boston Children's Hospital, Boston.

## Abstract

**Objective:**

Two cohort studies in patients with multisystem inflammatory syndrome in children (MIS‐C) demonstrated contrasting results regarding the benefit of initial immunomodulatory treatment with intravenous immunoglobulin (IVIG) alone versus IVIG and glucocorticoids. We sought to determine whether application of different MIS‐C definitions and differing disease severity between cohorts underlay discrepant results.

**Methods:**

The Overcoming COVID‐19 Public Health Surveillance Registry (OC‐19) included patients meeting the US Centers for Disease Control and Prevention (CDC) MIS‐C definition, whereas the Best Available Treatment Study (BATS) applied the World Health Organization (WHO) definition. We applied the WHO definition to the OC‐19 cohort and the CDC definition to the BATS cohort and determined the proportion that did not meet the alternate definition. We compared illness severity indicators between cohorts.

**Results:**

Of 349 OC‐19 patients, 9.5% did not meet the WHO definition. Of 350 BATS patients, 10.3% did not meet the CDC definition. Most organ system involvement was similar between the cohorts, but more OC‐19 patients had WHO‐defined cardiac involvement (87.1% vs 79.4%, *P* = 0.008). OC‐19 patients were more often admitted to intensive care (61.0% vs 44.8%, *P* < 0.001) and more often received vasopressors or inotropes (39.5% vs 22.9%, *P* < 0.001) before immunomodulatory treatment.

**Conclusion:**

Greater illness severity and cardiovascular involvement in the OC‐19 cohort compared with the BATS cohort, and not use of different MIS‐C case definitions, may have contributed to differing study conclusions about optimal initial treatment for MIS‐C. Disease severity should be considered in future MIS‐C study designs and treatment recommendations to identify patients who would benefit from aggressive immunomodulatory treatment.

## INTRODUCTION

Treatment of multisystem inflammatory syndrome in children (MIS‐C), a postacute inflammatory complication of severe acute respiratory syndrome coronavirus 2 (SARS‐CoV‐2) infection (1, 2), remains a pressing clinical issue as infections continue worldwide. Long‐term outcome data for patients with MIS‐C are still lacking. However, recent cohort studies presented contrasting results regarding short‐term outcomes of initial immunomodulatory treatment strategies (3, 4, 5). Results informed current clinical guidance, but different findings weakened evidence quality ratings (6, 7). Of the two largest studies, an analysis from the United States, the Overcoming COVID‐19 Public Health Surveillance Registry (OC‐19), found that initial treatment with intravenous immunoglobulin (IVIG) and glucocorticoids was associated with lower risk of cardiovascular dysfunction (shock requiring vasopressors or depressed left ventricular function) on or after 2 days of treatment compared with IVIG alone (adjusted risk ratio 0.56, 95% confidence interval [CI] 0.34‐0.94) (4). However, the international Best Available Treatment Study (BATS) found no statistically significant difference in odds of a composite outcome (death, receipt of inotropic or ventilatory support on day 2 or later) after initial treatment with IVIG alone or IVIG and glucocorticoids (adjusted odds ratio 0.77, 95% CI0.33‐1.82) (5). Primary outcomes were also similar for BATS patients initially treated with glucocorticoids alone.

Several factors have been proposed as contributing to these differing conclusions (8, 9, 10). First, OC‐19 included patients meeting the US Centers for Disease Control and Prevention (CDC) MIS‐C case definition (11), whereas BATS applied the World Health Organization (WHO) case definition (12). Both definitions, published in May 2020, include criteria regarding patient age, fever, systemic inflammation, multi–organ system involvement, positive laboratory testing results for SARS‐CoV‐2 or an epidemiologic link to COVID‐19, and lack of an alternative diagnosis (Table 1) (11, 12). However, these definitions differ with respect to patient age limits, fever duration, and organ system involvement criteria. These similar yet distinct definitions may have resulted in phenotypic differences in study patients, contributing to different conclusions about treatment efficacy. Second, application of these definitions may have varied by study sites. OC‐19 enrolled patients through active surveillance in US hospitals. In contrast, BATS recruited patients through passive surveillance, with 30% of patients from the United Kingdom, 10% from the United States, and 60% from 32 other countries. Third, MIS‐C illness severity may have been greater in OC‐19 patients compared with BATS patients (8, 9). If less severely ill patients were more likely to clinically recover without intervention, then differences in outcomes across treatment subgroups might have been attenuated.

**Table 1:** Comparison of MIS‐C criteria in the CDC and WHO case definitions as applied by the OC‐19 and BATS Consortium, respectively

Criterion	CDC MIS‐C case definition as applied to OC‐19 cohort	WHO MIS‐C case definition as applied to BATS cohort
Hospitalization	Required	Not required
No alternative diagnosis	Required	Required
Age	Less than 21 years	Less than 20 years
Fever (subjective report or temperature ≥38.0°C)	Duration of at least 24 hours	Duration of at least 3 days
Laboratory evidence of systemic inflammation	At least one of the following: CRP level ≥0.5 mg/dl, procalcitonin level ≥0.1 ng/ml, erythrocyte sedimentation rate >20 mm/hour, elevated ferritin level,^a^ fibrinogen level >400 mg/dl, D‐dimer level >500 ng/ml, absolute neutrophil count >8000/μl, absolute lymphocyte count <1000/μl	At least one of the following: CRP level ≥0.8 mg/dl, procalcitonin level ≥0.15 ng/ml
Evidence of SARS‐CoV‐2 infection	At least one of the following: contact with a case of COVID‐19 within the 4 weeks prior to symptom onset (accepted only for cases reported March 15 to May 31, 2020), positive SARS‐CoV‐2 serology test result, positive SARS‐CoV‐2 RT‐PCR result	At least one of the following: household contact with a confirmed or probable case of COVID‐19, positive SARS‐CoV‐2 serology test result, positive SARS‐CoV‐2 RT‐PCR result
Involvement of at least two organ systems or at least two clinical signs of multisystem involvement		
Cardiovascular or cardiac	At least one of the following: troponin levels elevated above hospital‐specific reference range,^b^ BNP or NT‐proBNP level >400 pg/ml, ventricular dysfunction, mitral or aortic regurgitation, pericardial effusion, coronary artery aneurysm,^c^ pericarditis or myocarditis, pulmonary edema due to left heart failure, arrhythmia, cardiac arrest, shock requiring vasopressors	At least one of the following: troponin level >0.014 ng/ml, BNP or NT‐proBNP level >400 pg/ml, left ventricular dysfunction, valvular dysfunction, pericardial effusion, coronary artery aneurysm,^d^ pericarditis or myocarditis, endocarditis^e^
Hypotension or shock	Shock requiring vasopressors included in cardiovascular	Receipt of intravenous fluid bolus^e^ or vasopressors
Coagulopathic	Not included	PT ≥14 seconds, INR ≥1.45, aPTT ≥40.4 seconds, or D‐dimer level ≥540 ng/ml
Hematologic	At least one of the following: thrombocytopenia, anemia, or leukopenia; venous thromboembolism; hemolysis^f^; bleeding^f^; ischemia of an extremity^f^	Not included
Gastrointestinal	At least one of the following: diarrhea, vomiting, abdominal pain, nausea or anorexia,^f^ hepatitis,^f^ gallbladder hydrops or edema,^f^ pancreatitis^f^	At least one of the following: diarrhea, vomiting, abdominal pain
Dermatologic or mucocutaneous	At least one of the following: rash or skin ulcers, mucous membrane changes, desquamation, conjunctivitis, peripheral extremity edema, erythema, or discoloration	At least one of the following: rash, mucous membrane changes, desquamation, conjunctivitis
Respiratory	At least one of the following: receipt of supplemental oxygen, lower respiratory infection, infiltrates on chest x‐ray,^f^ pleural effusion,^f^ bronchospasm,^f^ pulmonary hemorrhage,^f^ pneumothorax, receipt of chest tube, or thoracentesis^f^	Not included
Renal	Acute kidney injury or receipt of renal replacement therapy	Not included
Neurologic	At least one of the following: aseptic meningitis or encephalitis, seizure, stroke or intracranial hemorrhage, coma or unresponsiveness, receipt of neurodiagnostic testing,^f^ decreased vision or hearing,^f^ iritis or uveitis^f^	Not included

Abbreviations: aPTT, activated partial thromboplastin time; BATS, Best Available Treatment Study; BNP, brain natriuretic peptide; CDC, US Centers for Disease Control and Prevention; COVID‐19, coronavirus disease 2019; CRP, C‐reactive protein; INR, international normalized ratio; MIS‐C, multisystem inflammatory syndrome in children; NT‐proBNP, N‐terminal pro–brain natriuretic peptide; OC‐19, Overcoming COVID‐19 Public Health Surveillance Registry; PT, prothrombin time; RT‐PCR, reverse transcriptase polymerase chain reaction; SARS‐CoV‐2, severe acute respiratory syndrome coronavirus 2; WHO, World Health Organization.

^a^ Female patient aged <6 months: >375 ng/ml; female patient aged ≥6 months: >75 ng/ml; male patient aged <6 years: >75 ng/ml; male patient aged ≥6 years: >320 ng/ml.

^b^ Hospital‐specific troponin reference ranges were not collected by the BATS Consortium; a blanket threshold of 0.014 ng/ml was applied.

^c^ OC‐19 definition of coronary artery aneurysm: *z* score ≥2.5 of left anterior descending or right coronary artery.

^d^ BATS definition of coronary artery aneurysm: *z* score ≥2.5 or report of coronary artery aneurysm, excluding isolated left main coronary artery abnormality.

^e^ Data element not collected by OC‐19.

^f^ Data element not collected by BATS Consortium.

We aimed to further investigate reasons for differences in the estimated treatment effect by evaluating case definitions (11, 12), organ system involvement, and illness severity among patients included in the two cohort studies. We specifically evaluated severity of cardiovascular involvement because it directs MIS‐C management decisions and has been associated with mortality (6, 13).

## PATIENTS AND METHODS

### Patient enrollment and inclusion

Patients with MIS‐C were identified through active surveillance and included in OC‐19 after admission to 1 of 58 participating US hospitals. Cases were adjudicated by the site principal investigators and the coordinating center at Boston Children's Hospital with the CDC MIS‐C definition. The study was reviewed and approved by the Boston Children's Hospital Central Institutional Review Board. Informed consent was waived.

Data from 596 patients meeting the CDC definition were collected March 15 to October 31, 2020, and 349 patients were included in an inverse probability treatment weighting (IPTW) analysis comparing initial treatment (defined as first calendar day of immunomodulatory treatment) with IVIG alone versus IVIG and glucocorticoids (Supplementary Figure 1) (4). The results of this analysis supported those of the primary propensity score–matched analysis, which included 206 patients.

Patients in the BATS cohort, identified through passive surveillance, were hospitalized with suspected MIS‐C during June 20, 2020, to February 24, 2021. Data were submitted to a web‐based portal by clinicians at 81 hospitals in 34 countries. Clinicians at the coordinating site at Imperial College London adjudicated cases according to the WHO MIS‐C definition. The study was approved by the UK Health Research Authority and Research Ethics Committee (with reference 20/HRA/2957). Informed consent was waived. Participating centers obtained ethical approval according to requirements in each country.

Of 614 submitted patient reports, 414 were included in an IPTW analysis comparing initial treatment (defined as first calendar day of immunomodulatory treatment) with IVIG alone (n = 217) versus IVIG and glucocorticoids (n = 197); we included only the 350 (IVIG: n = 173, IVIG + glucocorticoids: n = 177) who met WHO MIS‐C criteria (Supplementary Figure 1) (5). Because patients initially treated with glucocorticoids alone were not included in OC‐19 analyses, BATS patients in this treatment group were excluded from the present analysis.

### Analysis

We compared patients meeting the CDC and WHO case definitions by applying the WHO definition to the OC‐19 cohort and applying the CDC definition to the BATS cohort as it was applied in OC‐19. In the primary analysis, the alternate definitions were applied to the cohorts included in the published OC‐19 and BATS IPTW analyses. In a secondary analysis, the definitions were also applied to the full OC‐19 cohort and all BATS patients meeting the WHO definition. When applying the WHO definition, we implemented minor changes from the way it was originally applied to the BATS cohort to more fully align with published WHO criteria (12): conjunctivitis and conjunctival injection were considered to fulfill the WHO mucocutaneous criteria, and elevations in brain natriuretic peptide (BNP) or N‐terminal pro‐BNP (NT‐proBNP) levels were considered to fulfill the WHO cardiac criteria. To align cohort definitions of coronary artery aneurysm, *z* scores of 2.5 or greater of the left main coronary artery were excluded. We calculated the proportion of patients in each cohort who met the alternate case definition and each of its components, including involvement of each organ system.

Indicators of general and cardiovascular illness severity were compared across the two cohorts. Pretreatment indicators included admission to an intensive care unit (ICU), receipt of vasopressors or inotropes, receipt of invasive mechanical ventilation, receipt of extracorporeal membrane oxygenation, and degree of elevation in C‐reactive protein, ferritin, troponin, BNP, and NT‐proBNP levels. Death at any time was also compared.

Fisher's exact test was used to compare dichotomous variables, and the Kruskal–Wallis test by ranks was used to compare laboratory values. Because fewer than half of OC‐19 patients had pretreatment measurement of troponin and natriuretic peptides, statistical comparison was not performed for these cardiac biomarkers.

Analyses were performed using R version 4.1.2. *P* values less than 0.05 were considered statistically significant for all tests.

## RESULTS

The WHO definition had more stringent requirements for age, fever duration, and laboratory evidence of inflammation, whereas the CDC definition required hospitalization, and OC‐19 required stricter evidence of SARS‐CoV‐2 infection, as laboratory evidence was required beginning June 1, 2020 (Table 1). The two definitions required dysfunction in at least two organ systems, but dysfunction in hematologic, respiratory, renal, and neurologic systems was unique to the CDC definition. The WHO definition, unlike the CDC definition, included a coagulopathic organ system and considered hypotension or shock distinct from other cardiovascular criteria. Differences in data collection between the two cohorts impeded precise application of the alternate case definition. For instance, intravenous fluid boluses were not recorded in OC‐19, limiting the identification of WHO‐defined hypotension, and chest radiograph findings were not recorded in BATS, limiting the identification of CDC‐defined respiratory criteria.

Of 349 OC‐19 patients, 33 (9.5%) did not meet the WHO definition (Supplementary Table 1). Although the CDC definition required 24 hours or more of fever, 331 (94.8%) OC‐19 patients had at least 3 days of fever as required by the WHO definition. Nearly all OC‐19 patients had WHO‐defined involvement of at least two organ systems (n = 341, 97.7%) and laboratory evidence of inflammation (n = 342, 98.0%). All met the WHO criteria for evidence of SARS‐CoV‐2 infection. Two OC‐19 patients (0.6%) were aged 20 years at hospitalization. Among the full OC‐19 cohort of 596 patients (including those excluded from the IPTW analysis), 16.3% did not meet the WHO definition (Supplementary Table 2).

Of 350 BATS patients, 36 (10.3%) did not meet the CDC definition (Supplementary Table 1). Most BATS patients (n = 318, 91.1%) met the CDC requirements for evidence of SARS‐CoV‐2 infection, and 98.6% (n = 345) had CDC‐defined involvement of at least two organ systems. Because age, fever, and inflammatory marker criteria were more stringent in the WHO definition, all BATS patients met these CDC criteria. Results were similar in the broader BATS subcohort of 484 patients who met the WHO definition (including those excluded from the IPTW analysis) (Supplementary Table 2).

**Figure 1:**
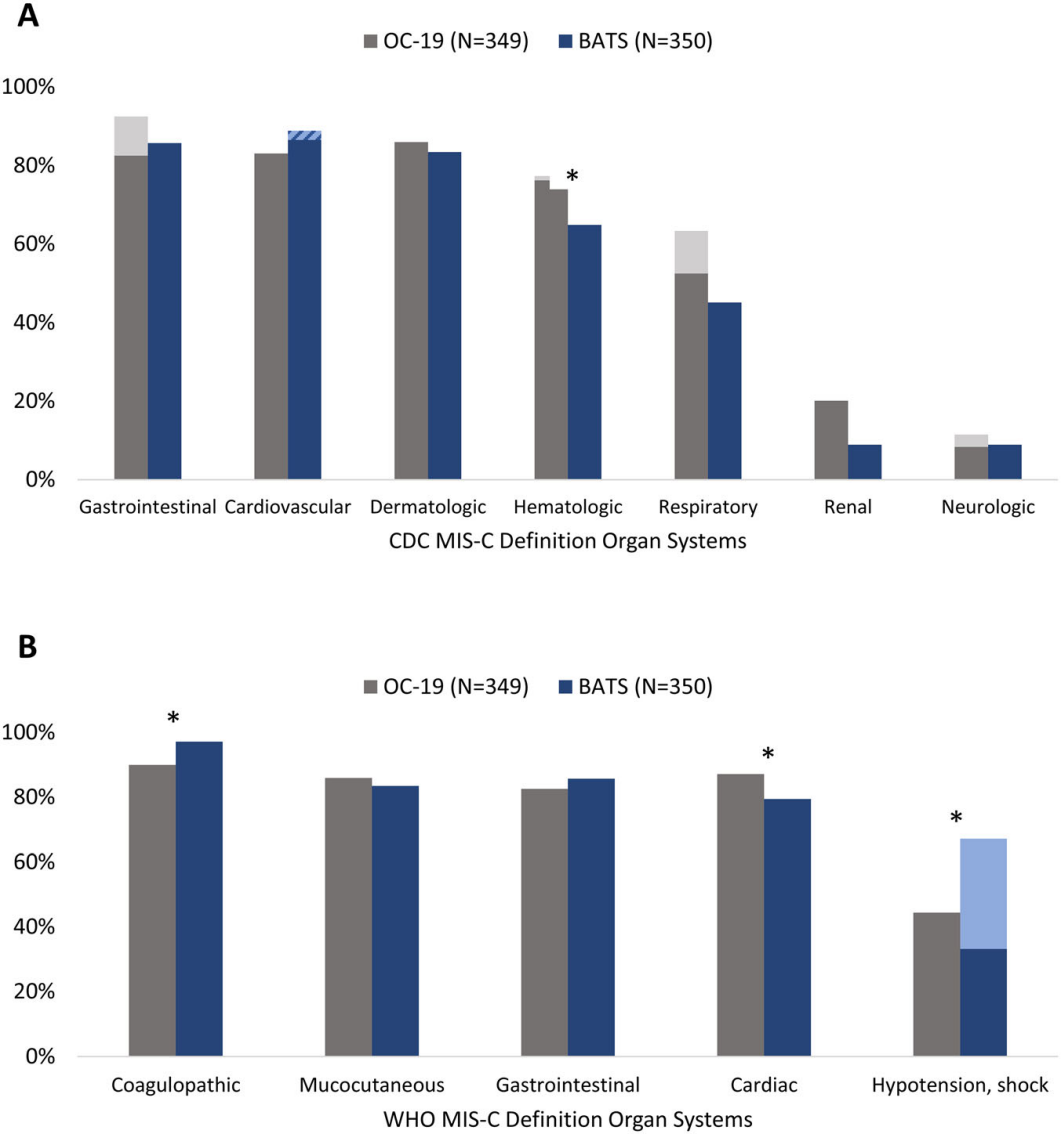
Organ system involvement among patients with multisystem inflammatory syndrome in children (MIS‐C) enrolled in the Overcoming Public Health Surveillance COVID‐19 Registry (OC‐19) and the Best Available Treatment Study (BATS) cohorts. Asterisks indicate statistically significant differences in organ system involvement across cohorts after we accounted for differences in data elements collected in the two cohorts. **A,** Organ system involvement per the US Centers for Disease Control and Prevention (CDC) MIS‐C case definition. Dark gray bars represent organ system involvement adjudicated using solely data elements available in BATS. Light gray bars represent organ involvement adjudicated using all OC‐19 data elements. The striped bar represents cardiovascular involvement adjudicated solely because of a troponin level of 0.014 to 0.03 ng/ml. BATS applied universal upper limit of normal 0.014 ng/ml. The median troponin upper limit of normal across OC‐19 enrollment sites was 0.03 ng/ml. **B,** Organ system involvement per the World Health Organization (WHO) case definition for MIS‐C. Dark blue bars represent organ system involvement adjudicated solely through data elements available in OC‐19. The light blue bar represents hypotension or shock identified either through vasopressor use or through intravenous fluid bolus administration. Intravenous fluid boluses were not recorded in OC‐19.

The CDC and WHO MIS‐C definitions included three of the same organ systems: cardiovascular or cardiac, gastrointestinal, and dermatologic or mucocutaneous (Table 1). When we accounted for differences in data elements collected in the cohorts, there were no significant differences in the proportions of OC‐19 and BATS patients who manifested the CDC criteria for these three organ systems (Figure 1). However, OC‐19 patients had higher frequency of WHO‐defined cardiac involvement (87.1% vs 79.4%, *P* = 0.008) and hypotension and shock (44.4% vs 33.1%, *P* = 0.002). When measured, median pretreatment troponin and BNP levels were higher among OC‐19 patients compared with BATS patients, whereas the median NT‐proBNP level was lower (Table 2). Of the organ systems unique to the CDC definition, OC‐19 patients were more likely to manifest hematologic (76.2% vs 64.9%, *P* = 0.001) and renal (20.1% vs 8.9%, *P* < 0.001) involvement. BATS patients were more likely to manifest coagulopathy, which is unique to the WHO definition (97.1% vs 90.0%, *P* < 0.001).

**Table 2:** Comparison of disease severity among patients with MIS‐C from the OC‐19 and BATS cohorts

	OC‐19 patients (n = 349)		BATS patients who meet the WHO case definition (n = 350)		
	Data available, n (%)	Result, n (%) or median (IQR)	Data available, n (%)	Result, n (%) or median (IQR)	*P*
Admission to an intensive care unit^a^	349 (100.0)	213 (61.0)	346 (98.9)	155 (44.8)	<0.001
Receipt of vasopressors or inotropes^a^	349 (100.0)	138 (39.5)	349 (100.0)	80 (22.9)	<0.001
Invasive mechanical ventilation^a^	349 (100.0)	20 (5.7)	350 (100.0)	23 (6.6)	0.75
Extracorporeal membrane oxygenation^a^	349 (100.0)	4 (1.1)	350 (100.0)	1 (0.3)	0.22
Hospital death	349 (100.0)	0 (0.0)	331 (94.6)	6 (1.8)	0.01
CRP^a^ (mg/dl)	289 (82.8)	15.4 (7.9‐23.4)	340 (97.1)	15.2 (9.3‐22.5)	0.64
Ferritin^a^ (ng/ml)	225 (64.5)	390 (220‐822)	301 (86.0)	444 (245‐774)	0.40
Troponin^a^ ^,^ ^b^ (ng/ml)	172 (49.3)	0.11 (0.02‐0.57)	252 (72.0)	0.04 (0.01‐0.15)	ND
BNP^a^ ^,^ ^b^ (pg/ml)	132 (37.8)	405 (79‐1071)	100 (28.6)	127 (43‐583)	ND
NT‐proBNP^a^ ^,^ ^b^ (pg/ml)	87 (24.9)	1106 (221‐2645)	138 (39.4)	1860 (468‐8137)	ND

Abbreviations: BATS, Best Available Treatment Study; BNP, brain natriuretic peptide; CRP, C‐reactive protein; IQR, interquartile range; MIS‐C, multisystem inflammatory syndrome in children; ND, not done; NT‐proBNP, N‐terminal pro–brain natriuretic peptide; OC‐19, Overcoming COVID‐19 Public Health Surveillance Registry; WHO, World Health Organization.

^a^ Pretreatment.

^b^ *P* values not calculated for biomarkers measured in <50% of patients in either cohort.

When we compared preimmunomodulatory treatment illness severity, more OC‐19 patients than BATS patients were admitted to intensive care (61.0% vs 44.8%, *P* < 0.001) and received vasopressors or inotropes (39.5% vs 22.9%, *P* < 0.001) (Table 2). There were no significant differences in proportions requiring invasive mechanical ventilation or extracorporeal membrane oxygenation. C‐ reactive protein and ferritin levels were similarly elevated in the two cohorts. None of the OC‐19 patients included in the propensity analysis died, compared with 1.8% of BATS patients (*P* = 0.01).

## DISCUSSION

Two observational treatment studies using MIS‐C cohorts, OC‐19 (CDC case definition) and BATS (WHO case definition), presented contrasting results regarding the relative benefit of initial immunomodulatory treatment modalities (4, 5). The OC‐19 analysis showed initial treatment with IVIG and glucocorticoids to be superior to treatment with IVIG alone, whereas the BATS analysis showed no statistically significant difference. Using the same study populations and examining factors that could have contributed to these contrasting results, we found that most patients in the analytic cohorts met both MIS‐C definitions. However, OC‐19 patients had greater pretreatment illness severity (as evidenced by more frequent ICU admission) and had greater severity of cardiovascular involvement (manifested by a higher proportion with shock requiring vasoactive agents), more WHO‐defined cardiac manifestations, and, when measured, greater elevation in troponin and BNP levels. As such, greater disease severity in the OC‐19 patient cohort likely contributed to detection of a treatment effect of adjunctive glucocorticoids with IVIG.

Greater pretreatment illness severity among patients in OC‐19, compared with those in BATS, is not explained by different MIS‐C definitions, nor can it be explained by differing levels of COVID‐19 vaccine uptake; vaccination was not yet recommended for most pediatric age groups at the time of either cohort assembly. Rather, it may be because OC‐19's participating institutions were tertiary referral centers and members of an existing pediatric critical care network (2). Although there were six deaths among BATS patients in the IPTW analysis and none among OC‐19 patients in the IPTW analysis, overall mortality in patients receiving primary treatment with IVIG or IVIG plus glucocorticoids is similar if two OC‐19 patients excluded for other reasons from the IPTW analysis are considered (4).

The main outcome in the OC‐19 IPTW analysis was more rapid resolution of cardiovascular instability, including left ventricular function impairment and shock requiring vasopressors. Similarly, a third MIS‐C observational treatment study among patients in France in which 90% required ICU admission and more than 50% required hemodynamic support before immunomodulatory therapy found that treatment initiation with IVIG and glucocorticoids, compared with IVIG alone, was associated with less hemodynamic support, cardiac ventricular dysfunction, and shorter duration of ICU stay (3). In adults with septic shock, early introduction of systemic glucocorticoids has been shown to lead to more rapid discontinuation of vasoactive agents for hypotension, although the effect on mortality is unclear (14). Careful analysis of glucocorticoid efficacy in MIS‐C is important because critically ill patients receiving glucocorticoids are at higher risk of complications, including hyperglycemia, gastrointestinal bleeding, and secondary infections.

MIS‐C is a rare complication of SARS‐CoV‐2 infection, and international trials may be needed to prospectively enroll sufficient patients to evaluate treatments and outcomes. Considering the impact of enrolling with CDC and/or WHO definitions is important. Differences in the definitions resulted from the urgent need in May 2020 to identify patients with MIS‐C, precluding harmonization, as well as the need for the WHO definition to be applicable in resource‐poor settings. Despite the differences, only 16% of OC‐19 patients with MIS‐C captured in the US registry did not meet the WHO definition, usually because of fewer than 3 days of fever. Only 10% of BATS patients who met the WHO definition did not meet the applied CDC definition, mostly because of epidemiologic linkage to a COVID‐19 case without laboratory evidence of current or prior SARS‐CoV‐2 infection. As such, despite differences in the two definitions, the organ systems affected in OC‐19 and BATS patients were similar, supporting a common set of MIS‐C manifestations, which may inform future definition revisions. Such revisions will also need to consider the impact of high population SARS‐CoV‐2 seroprevalence on interpretation of serologic testing over time. High COVID‐19 attack rates in the pediatric population during the omicron variant pandemic surge (15) and the wider introduction of COVID‐19 vaccines in childhood have already challenged interpretation of the requirement of laboratory or epidemiologic evidence of SARS‐CoV‐2 infection. A high proportion of children will have positive SARS‐CoV‐2 antibody testing results, which may complicate diagnosis of MIS‐C, particularly when alternative diagnoses, such as Kawasaki disease, are considered.

This study has limitations. First, comparisons across cohorts were challenged by lack of detail in the MIS‐C definitions, leading to differences in interpretation even when the definition components were equivalent. For instance, both definitions incorporate hypotension or shock (11, 12), but each investigator group implemented criteria for this feature differently. Hypotension ascertained through intravenous fluid bolus administration and vasoactive agents (in BATS) led to a higher proportion of patients than hypotension identified only through use of vasopressors (in OC‐19). Second, we could not account for differences in analytic methods (eg, selection of covariates for propensity scores) and in primary outcome definitions in the treatment studies, which may also have impacted study conclusions. Lastly, because BATS enrolled patients from multiple countries, there may have been differences in race and ethnicity and in underlying medical conditions between the two cohorts. Further, with potentially varying access to care, indicators of illness severity may not be directly comparable to the US cohort.

In summary, greater illness severity and cardiovascular involvement in the OC‐19 cohort compared with the BATS cohort, and not use of different MIS‐C case definitions, appears to contribute to differing study conclusions about optimal initial treatment for MIS‐C. In severe cases of MIS‐C with cardiovascular involvement, treatment with IVIG and glucocorticoids may lead to more rapid resolution of cardiovascular instability. Stratifying future MIS‐C treatment studies and recommendations according to disease severity or organ system involvement may better identify patients who benefit from more aggressive immunomodulatory treatment and improve patient outcomes.

## AUTHOR CONTRIBUTIONS

All authors were involved in drafting the article or revising it critically for important intellectual content, and all authors approved the final version to be published. Drs. Melgar and Son had full access to all of the data in the study and take responsibility for the integrity of the data and the accuracy of the data analysis.

### Study conception and design

Melgar, Seaby, McArdle, Campbell, Levin, Randolph, Son.

### Acquisition of data

Seaby, McArdle, Young, Levin, Randolph, Son.

### Analysis and interpretation of data

Melgar, Seaby, McArdle, Young, Campbell, Murray, Patel, Levin, Randolph, Son.

## Supporting information


Disclosuresform

**Appendix S1** Supplementary Information
