# Supplementary material for: Treatment of Multisystem Inflammatory Syndrome in Children: Understanding Differences in Results of Comparative Effectiveness Studies
Source: ACR Open Rheumatol. 2022 Jun 27;4(9):804–10. doi: 10.1002/acr2.11478 (PMC9469482; doi:10.1002/acr2.11478)
Supplement: Supplementary file 2 — Appendix S1 Supplementary Information [file ACR2-4-804-s002.docx]

**Supplemental Materials**

**The Best Available Treatment Study (BATS) Consortium**

The BATS consortium (<https://bestavailabletreatmentstudy.co.uk>) is composed, in alphabetical order for each country, by:

Imperial College London (Study Management group):
Study Coordinator: Professor Michael Levin
Co-investigators: Dr Claire Broderick, Dr Aubrey Cunnington, Dr Jethro Herberg, Dr Myrsini Kaforou, Dr Andrew McArdle, Dr Ruud Nijman, Dr Harsita Patel, Dr Eleanor Seaby, Dr Priyen Shah, Ms Ortensia Vito, Dr Elizabeth Whittaker, Dr Clare Wilson
Statisticians: Dr Clive Hoggart, Dr Myrsini Kaforou, Dr Andrew McArdle
Data management: Dr Tisham De, Ms Ortensia Vito

International Advisory Board:
Daniel Munblit^1^, Adriana Tremoulet^2^, Rolando Ulloa-Gutierrez^3^
^1^Department of Pediatrics and Pediatric Infectious Diseases, Institute of Child’s Health, Sechenov First Moscow State Medical University (Sechenov University), Moscow, Russia; ^2^Department of Pediatrics, Rady Children’s Hospital - San Diego, 3020 Children's Way, San Diego, CA 92123; ^3^Servicio de Infectología Pediátrica, Hospital Nacional de Niños "Dr. Carlos Sáenz Herrera", C.C.S.S., San José, Costa Rica

ARGENTINA
Jorge Agrimbau Vázquez^1^, Rodrigo Carmona^2^, Laura Pérez^3^, Mayra Rubiños^4^, Natalia Veliz^4^, Silvana Yori^1^
^1^ Department of Pediatrics, Outpatient Services, Hospital de Pediatría “Prof. Dr. Juan P. Garrahan”, Buenos Aires, Argentina
^2^ Pediatric Critical Care Unit, Hospital de Pediatría “Prof. Dr. Juan P. Garrahan”, Buenos Aires, Argentina
^3^ Department of Pediatrics, Emergency Medicine, Hospital de Pediatría “Prof. Dr. Juan P. Garrahan”, Buenos Aires, Argentina
^4^ Department of Pediatrics, Internal Medicine, Hospital de Pediatría “Prof. Dr. Juan P. Garrahan”, Buenos Aires, Argentina

AUSTRIA
Wolfgang Holter^1^, Matthias Krainz^1^, Raphael Ulreich^2^, Christoph Zurl^2^
^1^ St. Anna Children’s Hospital, Medical University of Vienna, Vienna, Austria
^2^ Department of Pediatrics, Pediatric Intensive Care Unit, Medical University Graz, Austria

BELGIUM
Filomeen Haerynck^1^, Levi Hoste^2^
^1^ Primary Immunodeficiency Research Lab, Center for Primary Immunodeficiency Ghent, Jeffrey Modell Diagnosis and Research Center, Ghent University Hospital, Ghent, Belgium
^2^ Department of Pediatric Pulmonology, Infectious Diseases and Immunology, Ghent University Hospital, Ghent, Belgium

BRAZIL
Izabel Alves Leal^4^, André Ricardo Araujo da Silva^2^, Anna Esther Araujo e Silva^1^,Andrea Barchik^4^, Sabrina T. A. Barreiro^1^, Natalia Cochrane^4^, Cristiane Henriques Teixeira^4^, Julienne Martins Araujo^1^, Rolando Andres Paternina-de la Ossa^3^, Cristina Souza Vieira^4^
^1^ Department of Pediatrics, Getulio Vargas Filho Hospital, Niteroi, RJ, Brazil
^2^ Materno Infantil Department, Federal Fluminense University, Brazil
^3^ Faculdade de Medicina de Ribeirāo Preto, Universidad de Sāo Paolo, Sāo Paolo, Brazil
^4^ Prontobaby Group, Rio de Janeiro, Brazil

BULGARIA
Anna Dimitrova^1^, Margarita Ganeva^1^, Stefan Stefanov^1^, Albena Telcharova-Mihaylovska^1^
^1^ Department of pediatric rheumatology, University Children’s Hospital, Medical University Sofia, Bulgaria

CANADA
Catherine M. Biggs^1^, Rosie Scuccimarri^2^, Davinia Withington^2^
^1^ Department of Pediatrics, The University of British Columbia, Vancouver, BC, Canada
^2^ Departments of Anesthesia and Pediatrics, Montreal Children's Hospital, McGill University Health Sciences Centre, Montreal, Quebec H4A 3J1, Canada

CHILE
Camila Ampuero^1^, Javiera Aravena^2^, Raul Bustos B^3^, Daniel Casanova^4^, Pablo Cruces^4,5,6^, Franco Diaz^4,6,7^, Tamara García-Salum^2^, Loreto Godoy^8^, Rafael A. Medina^2^, Gonzalo Valenzuela Galaz^2^
^1^ Unidad de Paciente Crítico, Hospital Clínico La Florida, Santiago, Chile
^2^ Department of Pediatric Infectious Diseases and Immunology, Pontifical Catholic University of Chile, Marcoleta 391, Santiago, Chile
^3^ Pediatric Intensive Care, Clínica Sanatorio Alemán, Concepción, Chile
^4^ Unidad de Paciente Crítico Pediátrico, Hospital de El Carmen de Maipú, Santiago, Chile
^5^ Centro de Investigación de Medicina Veterinaria, Escuela de Medicina Veterinaria, Facultad de Ciencias de la Vida, Universidad Andres Bello, Santiago, Chile
^6^ LARed Network, Santiago, Chile
^7^ Escuela de Medicina, Universidad Finis Terrae, Santiago, Chile
^8^ Unidad de Paciente Crítico Pediátrico (UPCP), Complejo Asistencial Dr. Sotero del Río, Santiago, Chile

COSTA RICA
María L. Avila-Aguero^1,4^, Helena Brenes-Chacón^1^, Gabriela Ivankovich-Escoto^2^, Rolando Ulloa-Gutierrez^1^, Adriana Yock-Corrales^1^
^1^ Servicio de Infectología Pediátrica, Hospital Nacional de Niños "Dr. Carlos Sáenz Herrera", C.C.S.S., San José, Costa Rica
^2^ Servicio de Immunología y Reumatología Pediátrica, Hospital Nacional de Niños "Dr. Carlos Sáenz Herrera", C.C.S.S., San José, Costa Rica
^3^ Servicio de Emergencias Pediátricas, Hospital Nacional de Niños "Dr. Carlos Sáenz Herrera", C.C.S.S., San José, Costa Rica
^4^ Center for Infectious Disease Modeling and Analysis (CIDMA), Yale University New Haven, Haven, CT, USA

EGYPT
Adham Badib^1^, Karim Badreldin^1^, Yara Elkhashab^1^, Hassan Heshmat^1^
^1^ Neonatal Intensive Care Unit, Smouha International Hospital, Alexandria, Egypt

FINLAND
Santtu Heinonen^1^
^1^ New Children's Hospital, Pediatric Research Center, University of Helsinki, and Helsinki University Hospital, 00029 HUS, Helsinki, Finland

FRANCE
François Angoulvant^1^,^2^, Alexandre Belot^3^, Naïm Ouldali^4,5,6^
^1^ Assistance Publique–Hôpitaux de Paris, Pediatric Emergency Department, Necker-Enfants Malades University Hospital, Université de Paris, Paris, France
^2^ INSERM, Centre de Recherche des Cordeliers, UMRS 1138, Sorbonne Université, Université de Paris, Paris, France
^3^ Hospices Civils de Lyon, Pediatric Nephrology, Rheumatology, Dermatology, Hopital Femme, Mère Enfant, Centre International de Recherche en Infectiologie/INSERM U1111, Bron, France
^4^ Assistance Publique–Hôpitaux de Paris, Department of General Pediatrics, Pediatric Infectious Disease and Internal Medicine, Robert Debré University Hospital, Université de Paris, Paris, France
^5^ ACTIV, Association Clinique et Thérapeutique Infantile du Val-de-Marne, Créteil, France
^6^ Université de Paris, INSERM UMR 1123, ECEVE, Paris, France

GERMANY
Florian Beske^1^, Axel Heep^1^, Katja Masjosthusmann^2^, Karl Reiter^3^, Ingeborg van den Heuvel^2^, Ulrich von Both^3^
^1^ Department of Pediatrics, University Hospital Oldenburg, Rahel-Straus-Str. 10, 26133 Oldenburg, Germany
^2^ University Hospital Muenster, Department of General Pediatrics, Albert-Schweizer-Campus A1, 48149 Muenster, Germany
^3^ University Hospital, Ludwig Maximilians University (LMU) Munich, Hauner Children's Hospital, Germany

GREECE
Aikaterini Agrafiotou^1^, Charalampos Antachopoulos^2^, Irini Eleftheriou^3^, Evangelia Farmaki^4^, Lampros Fotis^1,5,6^, Dimitrios Kafetzis^6^, Stavroula Lampidi^3^, Theodota Liakopoulou^1^, Despoina Maritsi^3^, Elisa Michailidou^2^, Maria Milioudi^2^, Ioanna Mparmpounaki^1^, Eleni Papadimitriou^2,4^, Vassiliki Papaevangelou^5^, Emmanuel Roilides^2^, Olga Tsiatsiou^2^, Georgios Tsolas^6^, Maria Tsolia^3^, Petrina Vantsi^2^
^1^ IASO Children's Hospital, Athens, Greece
^2^ Infectious Diseases Unit, 3rd Department of Pediatrics, School of Medicine, Faculty of Health Sciences, Aristotle University, Hippokration General Hospital, Thessaloniki, Greece
^3^ Second Department of Pediatrics, "P.& A. Kyriakou" Children's Hospital, Athens Medical School, National and Kapodistrian University of Athens, Greece
^4^ First Dept of Pediatrics, Hippokratio Hospital of Thessaloniki, Aristotle of Thessaloniki, Greece
^5^ ATTIKON General Hospital, Department of Pediatrics, National and Kapodsitrian University of Athens, Greece
^6^ Metropolitan General Hospital, Piraeus, Greece

HONDURAS
Linda Yajeira Banegas Pineda^1^, Karla Leversia Borjas Aguilar^2^, Edwin Mauricio Cantillano Quintero^1^
^1^ PICU, Department of Pediatric, North Hospital, IHSS, San Pedro Sula, Honduras
^2^ Pediatric Immunology Service, Hospital María, Especialidades Pediátricas, Tegucigalpa, Honduras

HONG KONG
Patrick Ip^1^, Mike Yat Wah Kwan^2^, Janette Kwok^4^, Yu Lung Lau^1^, Kelvin To^3^, Joshua Sung Chih Wong^2^
^1^ Department of Pediatrics and Adolescent Medicine, Li Ka Shing Faculty of Medicine, The University of Hong Kong, Hong Kong SAR, China
^2^ Pediatric Infectious Disease Unit, Hong Kong Hospital Authority Infectious Disease Center, Princess Margaret Hospital, Hong Kong SAR, China
^3^ Department of Microbiology, Li Ka Shing Faculty of Medicine, The University of Hong Kong, Hong Kong SAR, China
^4^ Department of Transplant and Immunogenetics, Queen Mary Hospital, Hong Kong SAR, China

HUNGARY
Mate David^1^, David Farkas^1^, Szofia Kalcakosz^1^, Klaudia Szekeres^1^, Borbala Zsigmond^1^
^1^ General Pediatrics, Heim Pal Children Hospital, ulloi ut 86. Budapest 1089, Hungary

INDIA
Nadeem Aslam^1^
^1^ Lotus Hospital for Women and Children, Pediatric ICU, Hyderabad, India

ITALY
Laura Andreozzi^5^, Francesco Bianco^1^, Valentina Bucciarelli^1^, Danilo Buonsenso^2^, Rolando Cimaz^3^, Patrizia D'Argenio^4^, Rosa Maria Dellepiane^3^, Marianna Fabi^5^, Maria Vincenza Mastrolia^6^, Angela Mauro^7^, Angelo Mazza^8^, Lorenza Romani^4^, Gabriele Simonini^6^, Vincenzo Tipo^7^, Piero Valentini^2^, Lucio Verdoni^8^
^1^ Department of Pediatric and Congenital Cardiology and Cardiac Surgery, Azienda Ospedaliero Universitaria “Ospedali Riuniti”, Ancona, Italy
^2^ Department of Woman and Child Health and Public Health, Fondazione Policlinico Universitario A. Gemelli IRCCS, Rome, Italy
^3^ University of Milano, Italy
^4^ Immunology and Infectious Diseases Unit, Academic Department of Pediatrics, Bambino Gesù Children's Hospital, IRCCS, Piazza Sant'Onofrio, 4, 00165 Rome, Italy
^5^ Department of Pediatrics, Sant'Orsola Malpighi University Hospital, Bologna, Italy
^6^ Rheumatology Unit, Meyer Children’s Hospital, University of Florence, Florence, Italy
^7^ Emergency Department, COVID Unit, Santobono-Pausilipon Children’s Hospital, Italy
^8^ Pediatric General Department, ASST Papa Giovanni XXIII, Bergamo, Italy

KENYA
Bhupi Reel^1^
^1^ MP Shah Hospital, Pediatric Intensive Care Unit, Nairobi, Kenya

MALTA
David Pace^1^, Paul Torpiano^1^
^1^ Department of Pediatric and Adolescent Health, Mater Dei Hospital, Malta

MEXICO
Marisol Fonseca Flores^1^, Miguel García Domínguez^2^, Ana Luisa Giron Vargas^1^, Liliana Lopez Hernández^1^, Roanne Patrician Mota Figueroa^1^, Giordano Pérez Gaxiola^2^, Julio Valadez^3^
^1^ Department of pediatric critical care, Centro Médico Nacional Siglo XXI, Mexico City, Mexico
^2^ Department of Allergy and Immunology, Hospital Pediatrico de Sinaloa, Culiacan, Mexico

NORWAY
Sjur Klevberg^1^, Per Kristian Knudsen^2^, Per Helge Måseide^1^
^1^ Children Department, Drammen Hospital, Vestre Viken Hospital Trust, Norway
^2^ Department of Pediatric Medicine, Oslo University Hospital, PB 4956 Nydalen, 0424 Oslo, Norway

PANAMA
Jose Manuel Carrera^1^, Elizabeth Castaño G^2^, Carlos Alberto Daza Timana^1^, Tirza De Leon^1^, Dora Estripeaut^3^, Jacqueline Levy Z^2^, Ximena Norero^4^, Javier Record^1^, Magda Rojas-Bonilla^5^
^1^ Department of Infectious Disease, Hospital Materno Infantil José Domingo De Obaldía, David, Chiriquí, Panamá
^2^ Pediatric Infectious Diseases Service, Hospital del Niño Dr. José Renán Esquivel, Panamá City, Panamá
^3^ Pediatric Infectious Diseases, Hospital Paitilla, Panamá, Panamá and Sistema Nacional de Investigación (SNI), SENACYT, Panamá
^4^ Pacifica Salud, Pediatric Infectious diseases, Panama City, Panama
^5^ Department of Pediatrics Infectious Diseases, Hospital de Especialidades Pediatricas, Panama, Republic of Panama

PARAGUAY
Ricardo Iramain^1^
^1^ Emergency Department, Hospital Clinicas-National University of Asuncion, Paraguay

PERU
Roger Hernandez^1,2^, Gian Huamán^1,2^, Manuel Munaico^1,2^, Carlos Peralta^1,2^, Diego Seminario^1,2^, Elmer Hans Zapata Yarlequé^1,2^
^1^ Departamento de pediatría, Hospital Cayetano Heredia, Lima, Perú
^2^ Servicio de pediatría, Clínica San Felipe, Lima, Perú

POLAND
Justyna Gadzinska^1^, Joanna Mandziuk^1^, Magdalena Okarska-Napierała^1^
^1^ Department of Pediatrics with Clinical Assessment Unit, Medical University of Warsaw, Żwirki i Wigury 63A Street, 02-091 Warsaw, Poland

RUSSIA
Zalina A. Alacheva^1^, Ekaterina Alexeeva^1,2^, Petr V. Ananin^1^, Margarita Antsupova^3^, Maya D. Bakradze^1^, Polina Bobkova^4^, Svetlana Borzakova^5,6^, Irina L. Chashchina^1^, Andrey P. Fisenko^1^, Marina S. Gautier^1^, Anastasia Glazyrina^7^, Elena Kondrikova^4^, Evgeniya Korobyants^7^, Anatoliy A. Korsunskiy^4^, Karina Kovygina^4^, Ekaterina Krasnaya^4^, Seda Kurbanova^7^, Maria K. Kurdup^1^, Anna V. Mamutova^1^, Lyudmila Mazankova^8^, Ilya L. Mitushin^1^, Daniel Munblit^4,9^, Anzhelika Nargizyan^7^, Yanina O. Orlova^1^,Ismail M Osmanov^3,5^, Anastasia S. Polyakova^1^, Olga Romanova^4^, Elmira Samitova^3,8^, Anna Sologub^7^, Ekaterina Spiridonova^4^, Rustem F. Tepaev^1,2^, Anna A. Tkacheva^1^, Valeriya Yusupova^7^, Elena Zholobova^10^
^1^ Infectious diseases hospital for children with COVID, National Medical Research Center for Children's Health, Moscow, Russia
^2^ Department of Pediatrics and Pediatric Rheumatology, Institute of Child’s Health, Sechenov First Moscow State Medical University (Sechenov University), Moscow, Russia
^3^ ZA Bashlyaeva Children’s Municipal Clinical Hospital, Moscow, Russia
^4^ Department of Pediatrics and Pediatric Infectious Diseases, Institute of Child’s Health, Sechenov First Moscow State Medical University (Sechenov University), Moscow, Russia
^5^ Pirogov Russian National Research Medical University, Moscow, Russia
^6^ Research Institute for Healthcare Organization and Medical Management of Moscow Healthcare Department, Moscow, Russia
^7^ Morozov Children's Municipal Clinical Hospital of the Moscow City Health Department, Moscow, Russia
^8^ Russian Medical Academy of Continuous Professional Education of the Ministry of Healthcare of the Russian Federation, Moscow, Russia
^9^ Inflammation, Repair and Development Section, National Heart and Lung Institute, Faculty of Medicine, Imperial College London, London, United Kingdom
^10^ Department of Children Diseases, Institute of Child’s Health, Sechenov First Moscow State Medical University (Sechenov University), Moscow, Russia

SPAIN
Carlos Daniel Grasa^1^, Nuria Lopez Segura^2^, Federico Martinon-Torres^3^, Susana Melendo^4^, Ana Méndez Echevarria^1^, Juan Miguel Mesa Guzmán^8,9^, Jorge Roberto Palacios Argueta^6^, Irene Rivero-Calle^3^, Jacques Rivière^4^, Moisés Rodríguez-González^7^, Pablo Rojo^5^, Judith Sánchez Manubens^6^, Pere Soler-Palacin^4^, Antoni Soriano-Arandes^4^, Alfredo Tagarro^5^, Serena Villaverde^5^
^1^ Department of Pediatric Infectious Diseases, Hospital Universitario La Paz, Madrid, 28046, Madrid, Spain
^2^ Hospital del Mar, Pediatrics, Spain
^3^ Translational Pediatrics and Infectious Diseases Department. Hospital Clínico Universitario de Santiago de Compostela. Av. Choupana, 15706 Santiago de Compostela, Spain
^4^ Pediatric Infectious Diseases and Immunodeficiencies Unit, Hospital Universitario Vall d’Hebron, Barcelona, Catalonia, Spain
^5^ Pediatric Research and Clinical Trials Unit (UPIC), Instituto de Investigación Sanitaria Hospital 12 de Octubre (IMAS12), Spain
^6^ Pediatric Cardiology Unit, Pediatric Medicine Service, Consorcio Sanitario Parc Tauli, Universitat Autonoma de Barcelona, Sabadell, Spain
^7^ Department of Pediatric Cardiology, Puerta del Mar University Hospital, Cadiz, Spain
^8^ Hospital Infanta Sofía, Pediatrics Department, San Sebastian de los Reyes, Madrid, Spain
^9^ Fundación para la Investigación e Innovación Biomédica de los Hospitales Infanta Sofía y Alcalá de Henares, Madrid, Spain

SWEDEN
Maria Altman^1^, Petter Brodin^2^, AnnaCarin Horne^3^, Karin Palmblad^3^
^1^ Clinical Epidemiology Division, Department of Medicine Solna, Karolinska University Hospital, Karolinska Institutet, Stockholm, Sweden
^2^ Science for Life Laboratory, Department of Women's and Children's Health, Karolinska Institutet, 17165, Solna, Sweden
^3^ Unit of Pediatric Rheumatology, Department of Women's and Children's Health, Karolinska Institutet, Karolinska University Hospital, Solna, 171 76, Stockholm, Sweden

SWITZERLAND
Barbara Brotschi^1^, Patrick Meyer Sauteur^1^, Jana Pachlopnik Schmid^1^, Seraina Prader^1^, Christa Relly^1^, Luregn J. Schlapbach^1^, Michelle Seiler^1^, Johannes Trück^1^, Daniela Wütz^1^
^1^ University Children's Hospital Zurich and Children's Research Center, University of Zurich (UZH), Switzerland

THE NETHERLANDS
Naomi Ketharanathan^1^, Clementien Vermont^1^
^1^ Department of Pediatric Infectious Diseases and Immunology, Erasmus MC-Sophia Children's Hospital, Rotterdam, The Netherlands

TURKEY
Esra Akyüz Özkan^1^, Emine Hafize Erdeniz^1^
^1^ Ondokuz Mayıs University Medical Faculty, Department of Pediatrics, Samsun, Turkey

UKRAINE
Galina Borisova^3^, Lidiya Boychenko^3^, Nadiia Diudenko^3^, Olexandr Kasiyan^2^, Kostiantyn Katerynych^4^, Kateryna Melnyk^3^, Nelia Miagka^3^, Maria Teslenko^4^, Mykola Trykosh^2^, Alla Volokha^1^
^1^ Shupyk National Medical Academy of Postgraduate Education, Kyiv, Ukraine
^2^ Kyiv City Children’s Clinical Hospital N°1, Kyiv, Ukraine
^3^ Kyiv City Children’s Clinical Hospital N°2, Kyiv, Ukraine
^4^ Medical Centre Dobrobut, Kyiv, Ukraine

UNITED KINGDOM
Toju Akomolafe^1^, Eslam Al-Abadi^17^, Nele Alders^2^, Paula Avram^19^, Alasdair Bamford^2^, Millie Banks^1^, Robin Basu Roy^1^, Thomas Beattie^3^, Olga Boleti^1^, Jonathan Broad^3^, Enitan D. Carrol^20^, Michael Carter^3^, Anchit Chandran^1^, Hannah Cooper^18^, Patrick Davies^16^, Marieke Emonts^5,6^, Ceri Evans^7^, Katy Fidler^15^, Caroline Foster^1^, Chen Gong^3^, Berin Gongrun^1^, Carmen Gonzalez^19^, Louis Grandjean^2^, Karlie Grant^2^, Yael Hacohen^2^, Jack Hall^1^, Jane Hassell^2^, Christine Hesketh^8^, Jessica Hewlett^3^, Ahmad Hnieno^1^, Hannah Holt-Davis^9^, Aleena Hossain^1^, Lee D Hudson^2^, Mae Johnson^2^, Sarah Johnson^1^, Deepthi Jyothish^18^, Beate Kampmann^10^, Akhila Kavirayani^11^, Deborah Kelly^12^, Filip Kucera^2^, Daniel Langer^8^, Jon Lillie^13^, Katherine Longbottom^1^, Hermione Lyall^1^, Niamh Mackdermott^1^, Sarah Maltby^19^, Thomas Mclelland^18^, Anne-Marie McMahon^19^, Danielle Miller^11^, Zoe Morrison^12^, Karyn Moshal^2^, Jennifer Muller^12^, Evangelia Myttaraki^1^, Simon Nadel^1^, Daniella Osaghae^1^, Fatima Osman^1^, Anna Ostrzewska^1^, Mrinalini Panthula^1^, Eleni Papachatzi^1^, Charalampia Papadopoulou^2^, Harsita Patel^1^, Justin Penner^2^, Shervin Polandi^1^, Andrew J. Prendergast^7^, Padmanabhan Ramnarayan^2^, Sophie Rhys-Evans^1^, Andrew Riordan^4^, Charlene M.C. Rodrigues^2^, Sam Romaine^20^, James Seddon^1^, Delane Shingadia^2^, Anand Srivastava^14^, Siske Struik^12^, Alice Taylor^2^, Amanda Taylor^1^, Andrew Taylor^18^, Steven Tran^1^, Gareth Tudor-Williams^1^, Fabian van der Velden^5,6^, Lyn Ventilacion^13^, Paul A Wellman^3^, Michael P. Yanney^14^, Shunmay Yeung^1^
^1^ Department of Pediatric Infectious Diseases, Imperial College Healthcare NHS Trust, London, W2 1NY, UK
^2^ Great Ormond Street Hospital for Children NHS Foundation Trust, London, WC1N 3JH, UK
^3^ Department of Women and Children's Health, School of Life Course Sciences, King's College London, St Thomas' Hospital, SE1 7EH, London, UK
^4^ Alder Hey Children's hospital, Pediatric Infectious Diseases, Liverpool, UK
^5^ Pediatric Immunology, Infectious Diseases and Allergy, Great North Children's Hospital, Newcastle upon Tyne Hospitals NHS Foundation Trust, Queen Victoria Road NE1 4LP, Newcastle upon Tyne, UK
^6^ Translational and Clinical Research Institute, Newcastle University, Newcastle upon Tyne, UK
^7^ Department of Pediatric Infectious Diseases, Royal London Hospital, Barts Health NHS Trust, London E1 1BB, UK and Blizard Institute, Queen Mary University of London, London E1 2AT, UK
^8^ Pediatric Emergency Department, St Helier Hospital, Wrythe Lane, Carshalton, Surrey, SM5 1AA, UK
^9^ Pediatric Department, West Cumberland Hospital, Whitehaven, CA28 8JG, UK
^10^ Faculty of Infectious and Tropical Disease, London School of Hygiene and Tropical Medicine, London, WC1E 7HT, UK
^11^ Department of Pediatric Rheumatology, Oxford University Hospitals NHS Foundation Trust, Oxford, OX3 7HE, UK
^12^ Children and Young Adult's Research Unit, Noah's Ark Children's Hospital for Wales, Heath Park, Cardiff, CF14 4XW, UK
^13^ Pediatric Department, Lister Hospital, East and North Hertfordshire Hospital NHS Trust, Stevenage, SG1 4AB, UK
^14^ Department of Pediatrics, Sherwood Forest Hospitals NHS Foundation Trust, UK
^15^ Department of Pediatrics, Royal Alexandra Children's Hospital, Eastern Road, Brighton East Sussex BN2 5BE, UK
^16^ Pediatric Critical Care Unit, Nottingham University Hospitals NHS Trust, Nottingham, UK
^17^ Childhood Arthritis and Rheumatic Diseases Unit, Birmingham Women's and Children's Hospital NHS FT, Birmingham, B4 6NH, UK
^18^ Department of General Pediatrics, Birmingham Women’s and Children’s NHS Foundation Trust, Birmingham, B4 6NH, UK
^19^ Pediatric Rheumatology Department, Sheffield Children's Hospital Western Bank, Sheffield, S10 2TH, UK
^20^ Institute of Infection Veterinary and Ecological Sciences, University of Liverpool, Liverpool, UK

UNITED STATES OF AMERICA
Aditya Badheka^1^, Sarah Badran^2^, Dwight M. Bailey^3^, Anna Kathryn Burch^4^, Jane C. Burns^5^, Catherine Cichon^5^, Blake Cirks^6^, Michael D. Dallman^7^, Dennis R. Delany^8^, Mary Fairchok^9^, Samantha Friedman^10^, Jennifer Geracht^6^, Allison Langs-Barlow^9^, Kelly Mann^9^, Amruta Padhye^10^, Alexis Quade^5^, Kacy Alyne Ramirez^14^, John Rockett^10^, Imran Ali Sayed^11^, Amr A. Shahin^12^, Adriana Tremoulet^5^, Samuel Umaru^13^, Rebecca Widener^4^
^1^ Department of Pediatrics, University of Iowa Stead Family Children’s Hospital, Iowa City, IA, 52242, USA
^2^ Cardiology, Children's Hospital Los Angeles, Los Angeles, CA, 90027, USA
^3^ Division of Pediatric Critical Care Medicine, Levine Children's Hospital, Atrium Health, Charlotte, NC, USA
^4^ Division of Infectious Disease, Department of Pediatrics. University of South Carolina School of Medicine, Prisma Health Children's Hospital - Midlands. Columbia, SC, 29203, USA
^5^ Department of Pediatrics, Rady Children’s Hospital - San Diego, 3020 Children's Way, San Diego, CA 92123, USA
^6^ Walter Reed National Military Medical Center, 8901 Wisconsin Avenue Bethesda Maryland, 20889-0001, USA
^7^ University of South Carolina School of Medicine, Department of Pediatrics, Division of Critical Care Medicine, Prisma Health Children’s Hospital – Midlands, Columbia, SC 29203, USA
^8^ Department of Pediatric Cardiology, Medical University of South Carolina, 10 McClennan Banks Drive, Charleston, SC, USA
^9^ Department of Pediatric Infectious Diseases M, Mary Bridge Children’s Hospital Tacoma, WA, USA
^10^ Pediatric Critical Care Division, University of Missouri Health Care, 400 N. Keene St., Columbia, Missouri, USA
^11^ Department of Pediatric Critical Care Medicine, University of Colorado, Children’s Hospital of Colorado at Colorado Springs, USA
^12^ Department of pediatric intensive care, Tucson Medical Center, Tucson, Arizona, USA
^13^ Pediatric Critical Care, Lehigh Valley Reilly Children's Hospital, Allentown, Pennsylvania, USA
^14^ Wake Forest Baptist Medical Center, Medical Center Blvd, Meads Hall, 3rd Floor, Department of Pediatrics Winston-Salem, North Carolina, 27157, USA

ZIMBABWE
Mujuru Hilda Angela^1^, Gwendoline Kandawasvika^1^
^1^ University of Zimbabwe, College of Health Sciences, Child and Adolescent Health Unit, Box A 168, Avondale, Harare, Zimbabwe

**Overcoming COVID-19 Investigators**

The following study group members were all closely involved with the design, implementation, and oversight of the Overcoming COVID-19 study.

Alabama: Children’s of Alabama, Birmingham. Michele Kong, MD.
Arizona: University of Arizona, Tucson. Mary Glas Gaspers, MD; Katri V. Typpo, MD.
Arkansas: Arkansas Children’s Hospital, Little Rock. Ronald C. Sanders Jr., MD, MS; Katherine Irby, MD.
California: Children’s Hospital of Orange County, Orange County. Adam J. Schwarz, MD.
California: Miller Children’s & Women’s Hospital Long Beach, Long Beach. Christopher J. Babbitt, MD.
California: UCSF Benioff Children’s Hospital Oakland, Oakland. Natalie Z. Cvijanovich, MD.
California: UCSF Benioff Children’s Hospital, San Francisco. Matt S. Zinter, MD.
Colorado: Children’s Hospital Colorado, Aurora. Aline B. Maddux, MD, MSCS; Peter M. Mourani, MD.
Connecticut: Connecticut Children’s, Hartford. Christopher L. Carroll, MD, MS.
Connecticut: Yale New-Haven Children’s Hospital, New Haven. John S. Giuliano, Jr., MD.
Florida: Holtz Children’s Hospital, Miami. Gwenn E. McLaughlin, MD, MSPH.
Georgia: Children's Healthcare of Atlanta at Egleston, Atlanta. Keiko M. Tarquinio, MD.
Illinois: Ann & Robert H. Lurie Children’s Hospital of Chicago, Chicago. Kelly N. Michelson, MD, MPH; Bria M. Coates, MD.
Indiana: Riley Hospital for Children, Indianapolis. Courtney M. Rowan, MD, MS.
Iowa: University of Iowa Stead Family Children’s Hospital, Iowa City. Kari Wellnitz, MD; Guru Bhoojhawon MBBS, MD.
Kentucky: University of Louisville and Norton Children’s Hospital, Louisville, Janice E. Sullivan, MD; Vicki L. Montgomery, MD; Kevin M. Havlin, MD.
Louisiana: Children's Hospital of New Orleans, New Orleans. Tamara T. Bradford, MD.
Maryland: Johns Hopkins Children’s Hospital, Baltimore. Becky J. Riggs,MD; Melania M. Bembea, MD, MPH, PhD.
Maryland: University of Maryland Children’s Hospital, Baltimore. Ana Lia Graciano, MD.
Maryland: Sinai Hospital of Baltimore, Baltimore. Susan V. Lipton, MD, MPH.
Massachusetts: Baystate Children’s Hospital, Springfield. Kimberly L. Marohn, MD.
Massachusetts: Boston Children’s Hospital, Boston. Adrienne G. Randolph, MD; Margaret M. Newhams, MPH; Sabrina R. Chen; Cameron C. Young; Suden Kucukak, MD; Katherine Kester; Jane W. Newburger, MD, MPH; Kevin G. Friedman, MD; Mary Beth F. Son, MD; Janet Chou, MD.
Massachusetts: MassGeneral Hospital for Children, Boston. Ryan W. Carroll, MD, MPH; Phoebe H. Yager, MD; Neil D. Fernandes, MBBS.
Michigan: Children’s Hospital of Michigan, Detroit. Sabrina M. Heidemann, MD.
Michigan: University of Michigan CS Mott Children’s Hospital, Ann Arbor. Heidi R. Flori, MD, FAAP.
Minnesota: University of Minnesota Masonic Children’s Hospital, Minneapolis, Janet R. Hume, MD, PhD.
Minnesota: Mayo Clinic, Rochester. Emily R. Levy, MD.
Mississippi: Children’s Hospital of Mississippi, Jackson. Charlotte V. Hobbs, MD.
Missouri: Children’s Mercy Hospital, Kansas City. Jennifer E. Schuster, MD.
Missouri: Washington University in St. Louis. Philip C. Spinella MD.
Nebraska: Children’s Hospital & Medical Center, Omaha. Melissa L. Cullimore, MD, PhD; Russell J. McCulloh, MD.
New Jersey: Hackensack University Medical Center, Hackensack. Katharine N. Clouser, MD.
New Jersey: Newark Beth Israel Medical Center, Newark. Rowan F. Walsh, MD.
New Jersey: Bristol-Myers Squibb Children's Hospital, New Brunswick. Lawrence C. Kleinman, MD, MPH, FAAP; Simon Li, MD, MPH; Steven M. Horwitz, MD.
New Jersey: St. Barnabas Medical Center, Livingston. Shira J. Gertz, MD.
New York: Golisano Children’s Hospital, Rochester. Kate G. Ackerman, MD; Jill M. Cholette, MD.
New York: Kings County Hospital, Brooklyn. Michael A. Keenaghan, MD.
New York: Maria Fareri Children's Hospital, Valhalla. Aalok R. Singh, MD.
New York: The Mount Sinai Hospital, New York City. Sheemon P. Zackai, MD; Jennifer K. Gillen, MD.
New York: Hassenfeld Children’s Hospital at NYU Langone, New York. Adam J. Ratner, MD, MPH; Heda Dapul, MD; Vijaya L. Soma, MD.
New York: Stony Brook University Hospital, Stony Brook. Ilana Harwayne-Gidansky, MD; Saul R. Hymes, MD.
New York: SUNY Downstate Medical Center University Hospital, Brooklyn. Sule Doymaz, MD.
North Carolina: University of North Carolina at Chapel Hill, Chapel Hill. Stephanie P. Schwartz, MD; Tracie C. Walker, MD.
Ohio: University Hospitals Rainbow Babies and Children's Hospital, Cleveland. Steven L. Shein, MD; Amanda N. Lansell, MD.
Ohio: Nationwide Children’s Hospital, Columbus. Mark W. Hall MD, FCCM.
Ohio: Cincinnati Children’s Hospital, Cincinnati. Mary A. Staat, MD, MPH.
Pennsylvania: Children’s Hospital of Philadelphia, Philadelphia. Julie C. Fitzgerald, MD, PhD, MSCE; Jenny L. Bush RN, BSN; Ryan H. Burnett, BS.
Pennsylvania: Penn State Children’s Hospital, Hershey. Neal J. Thomas, MD, MSc.
Pennsylvania: St. Christopher’s Hospital for Children, Philadelphia. Monica L. Koncicki, MD.
Pennsylvania: UPMC Children’s Hospital of Pittsburgh. Ericka L. Fink, MD, MS; Joseph A. Carcillo, MD.
South Carolina: MUSC Children’s Health, Charleston. Elizabeth H. Mack, MD, MS.; Laura Smallcomb, MD.
Tennessee: Monroe Carell Jr. Children’s Hospital at Vanderbilt, Nashville. Natasha B. Halasa, MD, MPH.
Tennessee: Le Bonheur Children’s Hospital, Memphis. Dai Kimura, MD.
Texas: Texas Children’s Hospital, Houston. Laura L. Loftis, MD.
Texas: University of Texas Health Science Center, Houston. Alvaro Coronado Munoz, MD.
Texas: University of Texas Southwestern, Children’s Medical Center Dallas, Dallas. Mia Maamari, MD; Cindy Bowens, MD, MSCS.
Utah: Primary Children’s Hospital, Salt Lake City. Hillary Crandall, MD, PhD.
Washington: Seattle Children’s Hospital, Seattle. Lincoln S. Smith, MD; John K. McGuire, MD.
CDC COVID-19 Response Team on Overcoming COVID-19: Manish M. Patel, MD, MPH; Leora R. Feldstein, PhD, MSc; Mark W. Tenforde, MD PhD; Ashley M. Jackson MPH; Nancy Murray MSc; Charles E. Rose, PhD.

**
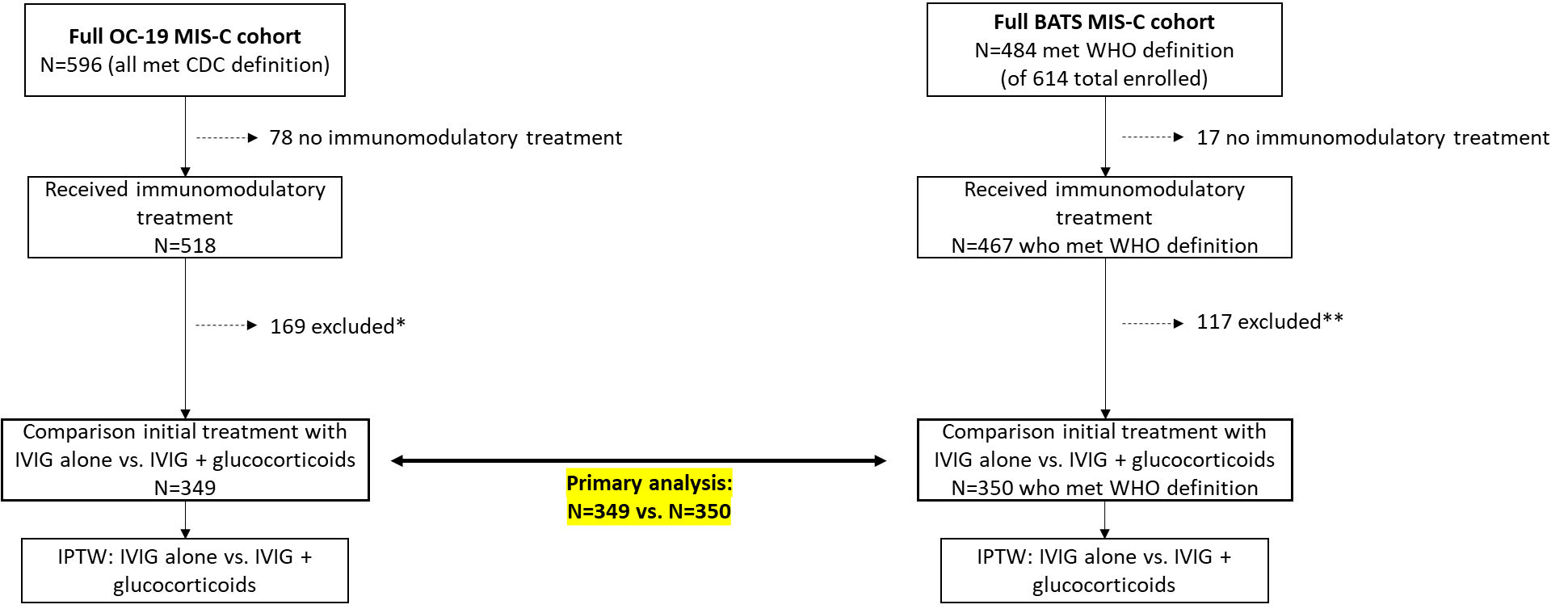
**

**Figure S1.** Inclusion in Overcoming COVID-19 and BATS treatment effectiveness studies and in the present study.

*169 excluded from OC-19 cohort due to initial treatment other than IVIG alone or IVIG + glucocorticoids, missing treatment dates, or other reasons [Son MBF, Murray N, Friedman K, Young CC, Newhams MM, Feldstein LR, et al. Multisystem Inflammatory Syndrome in Children - Initial Therapy and Outcomes. N Engl J Med. 2021;385(1):23-34]

**117 excluded from BATS cohort due to initial treatment before transfer to reporting hospital or initial treatment other than IVIG alone or IVIG + glucocorticoids [McArdle AJ, Vito O, Patel H, Seaby EG, Shah P, Wilson C, et al. Treatment of Multisystem Inflammatory Syndrome in Children. N Engl J Med. 2021;385(1):11-22]

**Table S1.** CDC and WHO MIS-C case definition components not met among patients in the Overcoming COVID-19 MIS-C cohort and the BATS cohort, restricted to participants included in inverse probability treatment weighted analyses.

|  | **Proportion of OC-19 participants (n=349) who did NOT meet criterion** | | **Proportion of BATS participants (n=350) who did NOT meet criterion** | |
| --- | --- | --- | --- | --- |
|  | **n** | **%** | **n** | **%** |
| **Full CDC case definition** |  |  | **36** | **10.3** |
| Illness requiring hospitalization |  |  | 0 | 0.0 |
| Age <21 years |  |  | 0 | 0.0 |
| Fever (subjective or measured^a^) for ≥24 hours |  |  | 0 | 0.0 |
| Involvement of ≥2 organ systems |  |  | 5 | 1.4 |
| Laboratory evidence of inflammation |  |  | 0 | 0.0 |
| No alternative diagnoses |  |  | 0 | 0.0 |
| Laboratory test positive for current or recent SARS-CoV-2 infection or COVID-19 exposure within the 4 weeks prior to onset of symptoms |  |  | 32 | 9.1 |
|  |  |  |  |  |
| **Full WHO case definition** | **33** | **9.5** |  |  |
| Age 0–19 years | 2 | 0.6 |  |  |
| Fever (subjective or measured^a^) for ≥3 days | 18 | 5.2 |  |  |
| Involvement of ≥2 organ systems | 8 | 2.3 |  |  |
| Laboratory evidence of inflammation | 7 | 2.0 |  |  |
| No alternative diagnoses | 0 | 0.0 |  |  |
| Laboratory evidence of COVID-19 or household contact with a case of COVID-19 | 0 | 0.0 |  |  |

Abbreviations: BATS, Best Available Treatment Study; CDC, U.S. Centers for Disease Control and Prevention; COVID-19, coronavirus disease 2019; MIS-C, multisystem inflammatory syndrome in children; OC-19; Overcoming COVID-19; SARS-CoV-2, severe acute respiratory syndrome coronavirus 2; WHO, World Health Organization
^a^ Temperature ≥38.0° C

**Table S2.** CDC and WHO MIS-C case definition components not met among patients in the Overcoming COVID-19 MIS-C cohort and the BATS cohort, including all Overcoming COVID-19 participants and all BATS participants who met the WHO definition.

|  | **Proportion of OC-19 participants (n=596) who did NOT meet criterion** | | **Proportion of BATS participants (n=484) who did NOT meet criterion** | |
| --- | --- | --- | --- | --- |
|  | **n** | **%** | **n** | **%** |
| **Full CDC case definition** |  |  | **52** | **10.7** |
| Illness requiring hospitalization |  |  | 0 | 0.0 |
| Age <21 years |  |  | 0 | 0.0 |
| Fever (subjective or measured^a^) for ≥24 hours |  |  | 0 | 0.0 |
| Involvement of ≥2 organ systems |  |  | 9 | 1.9 |
| Laboratory evidence of inflammation |  |  | 0 | 0.0 |
| No alternative diagnoses |  |  | 0 | 0.0 |
| Laboratory test positive for current or recent SARS-CoV-2 infection or COVID-19 exposure within the 4 weeks prior to onset of symptoms |  |  | 45 | 9.3 |
|  |  |  |  |  |
| **Full WHO case definition** | **97** | **16.3** |  |  |
| Age 0–19 years | 7 | 1.2 |  |  |
| Fever (subjective or measured^a^) for ≥3 days | 50 | 8.4 |  |  |
| Involvement of ≥2 organ systems | 29 | 4.9 |  |  |
| Laboratory evidence of inflammation | 29 | 4.9 |  |  |
| No alternative diagnoses | 0 | 0.0 |  |  |
| Laboratory evidence of COVID-19 or household contact with a case of COVID-19 | 0 | 0.0 |  |  |

Abbreviations: BATS, Best Available Treatment Study; CDC, U.S. Centers for Disease Control and Prevention; COVID-19, coronavirus disease 2019; MIS-C, multisystem inflammatory syndrome in children; OC-19; Overcoming COVID-19; SARS-CoV-2, severe acute respiratory syndrome coronavirus 2; WHO, World Health Organization
^a^ Temperature ≥38.0° C
